# Distributed Space Debris Tracking with Consensus Labeled Random Finite Set Filtering †

**DOI:** 10.3390/s18093005

**Published:** 2018-09-07

**Authors:** Baishen Wei, Brett Nener

**Affiliations:** 1 School of Mechanical and Electric Engineering, Guangzhou University, Guangzhou 510006, China; 2 School of Electrical, Electronic, and Computer Engineering, the University of Western Australia, Crawley, WA 6009, Australia; brett.nener@uwa.edu.com

**Keywords:** space debris, marginalized *δ*-GLMB, distributed, consensus

## Abstract

Space debris tracking is a challenge for spacecraft operation because of the increasing number of both satellites and the amount of space debris. This paper investigates space debris tracking using marginalized δ-generalized labeled multi-Bernoulli filtering on a network of nodes consisting of a collection of sensors with different observation volumes. A consensus algorithm is used to achieve the global average by iterative regional averages. The sensor network can have unknown or time-varying topology. The proposed space debris tracking algorithm provides an efficient solution to the key challenges (e.g., *detection uncertainty*, *data association uncertainty*, *clutter*, etc.) for space situational awareness. The performance of the proposed algorithm is verified by simulation results.

## 1. Introduction

This paper presents a distributed space objects tracking approach in the context of space situational awareness (SSA) [1]. SSA is the ability to track and predict the velocity and location of space objects in orbit around the Earth. SSA has become a significant concern for both commercial and military systems. A U.S. Iridium 33 communications satellite was struck by a defunct Russian Cosmos 2251 military communications satellite in February 2009. Collisions like this generate space debris, which then increases the likelihood of further collisions [2]. Collisions may degrade the performance of a spacecraft or even fragment one if they involve enough energy [3]. Multi-object tracking algorithms play a significant role in space object tracking [4,5].

The catalog size which future space objects tracking systems should be able to handle is more than 100,000 resident space objects [6]. A possible approach to tracking space debris is by the use of a network with regional nodes. Multi-nodal systems which consist of multiple agents with sensing, processing, and communication capabilities are widely used in multiple object tracking systems. Information fusion methods used in multi-node systems fall into three categories: distributed, centralized, and hierarchical. The Joint Space Operations Center (JSpOC) currently employs the centralized information processing approach because it can provide the most accurate estimation [6]. As the space debris tracking system has more nodes, the computation load of centralized systems becomes very challenging. Distributed information fusion can be used as an alternative solution [7]. The main advantages of distributed systems include: the system is reliable (i.e., resilient to failures), the network topology can be time-varying or unknown, and the processing is scalable with the number of nodes [8].

A consensus approach can be used in a distributed system to achieve global information fusion over the network [9,10]. Consensus has emerged as a useful tool for parameter estimation and distributed information fusion [11,12]. The logic behind consensus is to reach a global average by allowing each node to perform iterative local averages [13]. Global average and local average are achieved with information fusion over the whole network and neighboring nodes, respectively. The information can be propagated throughout the entire network with such repeated local information fusion [14,15]. The computation load for each node only depends on the number of its neighboring nodes. The calculation for each node does not increase dramatically with the number of nodes in the whole network [16,17]. A consensus approach for multi-target tracking with labeled random finite set (RFS) filtering was presented in [18]. The Kullback–Leibler average for probability densities can be used to achieve consensus for distributed estimation in a general state space model [19].

The scenarios presented in most of the information fusion literature have the same observation volume, with objects staying inside the observation volume all the time. However, space debris does not stay inside the observation volume except for those in geostationary equatorial orbit. Normal multi-object filtering methods do not provide estimation for objects outside the observation volume, and fusion among sensors with different observation volumes fails to provide accurate results.

Multi-object tracking involves the estimation of an unknown number of objects and their trajectories that are time-varying from noisy observations. The RFS approach has been widely used in multi-object tracking. The multi-object state is modeled as an RFS, and a systematic treatment of a multi-object system is provided [20,21]. Due to the complexity of the Bayes filter, the probability hypothesis density (PHD) [22,23], cardinalized PHD (CPHD) [24,25], and multi-Bernoulli filters [26,27] have been proposed as approximations. An analytic solution, the δ-generalized labeled multi-Bernoulli (δ-GLMB) filter and its efficient implementation were proposed in [28,29], respectively. Another efficient implementation with joint prediction and update and Gibbs sampling is detailed in [30]. Marginalized δ-GLMB (Mδ-GLMB) filter and labeled multi-Bernoulli (LMB) filter were proposed in [31,32], respectively, as two efficient approximations.

Most SSA literature treats the core elements of data association, detection, and tracking separately. An integrated approach to all these problems can limit the information loss and improve tracking performance. The δ-GLMB filter can provide object trajectories in the presence of missed detection, clutter, and association uncertainty [28]. It is an exact closed-form solution to multi-object Bayes recursion. The number of components grows without bound in time because of the explicit data associations in the filter. Mδ-GLMB is an efficient approximation to the δ-GLMB filter which preserves both the cardinality distribution and the PHD of the posterior [31]. Moreover, Mδ-GLMB densities are algebraically closed under Kullback–Leibler averaging, which makes Mδ-GLMB filtering a good choice in distributed processing using a consensus approach [18].

The space debris tracking problem has been an active research area [33,34,35,36,37,38,39,40]. A space object tracking approach with CPHD filtering and a measurement-based birth model was presented in [41]. A labeled multi-Bernoulli filter for space object tracking was proposed in [4]. GM-CPHD filtering and a consensus algorithm were used in [42] to achieve global space object tracking. A consensus algorithm was used for distributed information fusion. Even though estimation performance was promising, object trajectories were not available. Preliminary results to track space debris with consensus LMB filtering was proposed in [40], which can only provide state estimation for targets inside of the observation volume and fuse information from sensors with the same observation volume.

This paper proposes a consensus Mδ-GLMB approach for space debris tracking. Sensors can have the same or different observation volumes. Iterative consensus is performed among neighboring nodes to achieve a global average over the sensor network, which makes the fusion scalable with respect to the number of nodes. The topology of the sensor network can be time-varying. The network is resilient to failure since there is no coordination node. In order to achieve space object tracking within a single framework, the software Turboprop [43] and UKF are used to approximate the transition density of space objects. Turboprop has a package of functions which can be used to calculate the trajectories of space debris.

The main contribution of this paper is the distributed fusion algorithm for sensors with different observation volumes. Most fusion methods assume that all sensors have the same observation volume or that targets stay in the combined observation volume all the time. When targets leave the observation volume, normal algorithms cannot keep the estimation of targets because there are no measurements available. In this paper, the detection probability outside of the observation volume is set to zero. The expectation maximization (EM) algorithm is used to approximate the densities across the observation volumes. Since there are no measurements for targets outside the observation volume, the estimation is essentially the prediction. Then, the fusion among sensors with different observation volumes can be performed in the same way as fusion among sensors with the same observation volume.

The paper is organized as follows. Background on labeled RFS and the network model are described in Section 2. The Mδ-GLMB filter recursion is presented in Section 3. The space debris dynamic model is provided in Section 4. Section 5 details the estimation for objects when they are outside the observation volume. Section 6 details the consensus with Mδ-GLMB filtering. Numerical results are presented in Section 7, and concluding remarks are given in Section 8.

## 2. Background

### 2.1. Notation

In this paper, the Kronecker delta that takes arbitrary arguments is denoted by:

(1) $$\begin{array}{c} \delta_{Y}\left(X\right)≜\left\{\begin{array}{c} 1,\mathrm{if}\;\;\;X=Y,\\ 0,\mathrm{otherwise}. \end{array}\right. \end{array}$$

The inclusion function is denoted by:

(2) $$\begin{array}{c} 1_{Y}\left(X\right)≜\left\{\begin{array}{c} 1,\mathrm{if}\;\;\;X\subseteq Y,\\ 0,\mathrm{otherwise}. \end{array}\right. \end{array}$$

The standard inner product notation is denoted by 〈f,g〉≜∫f(x)g(x)dx, and the exponential notation $h^{X}≜\Pi_{x\in X}h\left(x\right)$, where *h* is a real-valued function. The weighting operator ⊙ for a given probability density function (PDF) *p* and a scalar α is defined as:

(3) $$\begin{array}{c} \left(\alpha ⊙p\right)\left(x\right)≜\frac{{\left[p\left(x\right)\right]}^{\alpha }}{\langle p^{\alpha },1\rangle }. \end{array}$$

### 2.2. Network Model

The nodal network can be denoted by a directed graph G=(M,A). M denotes the set of nodes and A=M×M denotes the connections among nodes. (i,j)∈A if node *j* can receive data from node *i*. $M^{\left(j\right)}≜\left\{i\in M:\left(i,j\right)\in A\right\}$ represents the set of neighbors for each node j∈M (including *j* itself).

Every node in the network has the same importance and performs the same actions: gathers measurements, carries out local computation, and exchanges information with neighbors. The fusion is performed among its own information and information from its neighbors.

### 2.3. Labeled RFS and Bayesian Multi-Object Filtering

An RFS is a finite set valued random variable. A unique label $\ell \in L=\{\alpha_{i}:i\in N\}$ is added to the dynamic state x∈X to incorporate the identity of objects, where N is the set of positive integers and the $\alpha_{i}$’s are distinct. Labels for objects are ordered pairs of integers ℓ=(k,i), where *k* is the time of object birth and i∈N is the index to distinguish objects born at the same time. $L_{k}=\left\{k\right\}\times N$ is used to denote the label space for objects born at time *k*. Then, the new object born at time *k* has the state $x\in X\times L_{k}$. The label space can be constructed recursively by $L_{0:k}=L_{0:k-1}\cup L_{k}$. The abbreviation $L≜L_{0:k}$ is used for compactness.

Let L:X×L→L denote the projection L((x,ℓ))=ℓ, then the function $\Delta (X)≜\delta_{\left|X\right|}\left(|L\left(X\right)|\right)$ is called the *distinct label indicator*, which means that X has the same cardinality as its labels L(X)={L(x):x∈X}. The labeled RFS can be unlabeled by discarding the labels. Let the labeled RFS distribution be denoted by $\pi (\left\{\left(x_{1},\ell_{1}\right),\ldots ,\left(x_{n},\ell_{n}\right)\right\})$. Then, the unlabeled RFS is distributed according to:

$$\begin{array}{c} \pi \left(\left\{x_{1},\ldots ,x_{n}\right\}\right)=\sum_{\left(\ell_{1},\ldots ,\ell_{n}\right)\in L^{n}}\pi \left(\left\{\left(x_{1},\ell_{1}\right),\ldots ,\left(x_{n},\ell_{n}\right)\right\}\right). \end{array}$$

The cardinality distribution (the distribution of the number of objects) of a labeled RFS is the same as its unlabeled version. More information about labeled RFSs can be found in [28,29].

Assume that there are N(k) object states $x_{k,1},\ldots ,x_{k,N(k)}$ with state space X×L at time *k*, and M(k) measurements $z_{k,1},\ldots ,z_{k,M(k)}$ with observation space Z. Then, the set of objects and observations are treated as the *multi-object state* and *multi-object observation*, respectively:

$$\begin{array}{ccc} X_{k} & = & \{x_{k,1},\ldots ,x_{k,N(k)}\},\\ Z_{k} & = & \{z_{k,1},\ldots ,z_{k,M(k)}\}. \end{array}$$

Let $\pi_{k|k-1}$ denote the *multi-object prediction density* and $\pi_{k}\left(\cdot |Z_{k}\right)$ denote the *multi-object filtering density* at time *k*. Then, the object density is propagated by the *multi-object Bayes filter* in time according to:

(4) $$\begin{array}{ccc} \pi_{k|k-1}\left(X_{k}\right) & = & \int f_{k|k-1}\left(X_{k}|X_{k-1}\right)\pi_{k-1}\left(X_{k-1}|Z_{k-1}\right)\delta X_{k-1}, \end{array}$$

(5) $$\begin{array}{ccc} \pi_{k}\left(X_{k}|Z_{k}\right) & = & \frac{g_{k}\left(Z_{k}|X_{k}\right)\pi_{k|k-1}\left(X_{k}\right)}{\int g_{k}\left(Z_{k}|X_{k}\right)\pi_{k|k-1}\left(X_{k}\right)\delta X}, \end{array}$$

where $f_{k|k-1}\left(\cdot |\cdot \right)$ is the *multi-object transition density* to time *k* and $g_{k}\left(\cdot |\cdot \right)$ is the *multi-object likelihood function* at time *k*. The underlying models of object births, deaths, and motions are encapsulated in the multi-object transition density, while the underlying models for detections and false alarms are encapsulated in the multi-object likelihood function.

For convenience, we omit the references to the time index *k* and denote $g≜g_{k}$, $f≜f_{k|k-1}$, $\pi_{+}≜\pi_{k|k-1}$, $\pi ≜\pi_{k}$, $B≜L_{k}$, $L_{+}≜L\cup B$.

### 2.4. δ-Generalized Labeled Multi-Bernoulli

The δ-GLMB density has the form

(6) $$\begin{array}{c} \pi \left(X\right)=\Delta \left(X\right)\sum_{(I,\xi )\in F(L)\times \Xi }\omega^{\left(I,\xi \right)}\delta_{I}\left(L\left(X\right)\right){\left[p^{\left(\xi \right)}\right]}^{X}, \end{array}$$

where each *I* represents a set of track labels, each ξ represents a history of association maps, and Ξ is a discrete space. The pair (I,ξ)∈F(L)×Ξ is called a *hypothesis* and the associated weight $\omega^{\left(I,\xi \right)}$ means the probability of the hypothesis. $p^{\left(\xi \right)}$ is the density of the kinematic state [28,29].

## 3. The Mδ-GLMB Filter Recursion

Mδ-GLMB filter is an approximation to the δ-GLMB filter. It can be interpreted as performing a marginalization over the association histories. The Mδ-GLMB filter has the same cardinality distribution and PHD as the δ-GLMB filter [31].

An Mδ-GLMB density corresponding to the δ-GLMB density in (6) is of the form

(7) $$\begin{array}{c} \pi \left(X\right)=\Delta \left(X\right)\sum_{I\in F(L)}\omega^{\left(I\right)}\delta_{I}\left(L\left(X\right)\right){\left[p^{\left(I\right)}\right]}^{X}, \end{array}$$

where

(8) $$\begin{array}{ccc} \omega^{\left(I\right)} & = & \sum_{\xi \in \Xi }\omega^{\left(I,\xi \right)}, \end{array}$$

(9) $$\begin{array}{ccc} p^{\left(I\right)}\left(x,\ell \right) & = & 1_{I}\left(\ell \right)\frac{1}{\omega^{\left(I\right)}}\sum_{\xi \in \Xi }\omega^{\left(I,\xi \right)}p^{\left(\xi \right)}\left(x,\ell \right). \end{array}$$

A Mδ-GLMB is a special case of the δ-GLMB, and it is completely characterized by the parameter set $\pi =\{\left(\omega^{\left(I\right)},p^{\left(I\right)}\right):I\in F\left(L\right)\}$. The Mδ-GLMB prediction and update are outlined below.

### 3.1. Mδ-GLMB Prediction

For the current time and a given multi-object state X, each state (x,ℓ)∈X either survives with probability $p_{S}\left(x,\ell \right)$ and evolves to the next step with new state $\left(x_{+},\ell_{+}\right)$, or dies with probability $1-p_{S}\left(x,\ell \right)$. The birth density of the new objects at the next time step is

(10) $$\begin{array}{c} f_{B}\left(Y\right)=\Delta \left(Y\right)\omega_{B}\left(L\left(Y\right)\right){\left[p_{B}\right]}^{Y}, \end{array}$$

where $p_{B}$ and $\omega_{B}$ are given parameters of the birth density.

The multi-object state at the next time step $X_{+}$ includes two parts: the new-born objects and the surviving objects. Supposing that the births are independent of surviving objects and that objects evolve independently of each other, the multi-object transition density is given by [28,29]:

(11) $$\begin{array}{c} f\left(X_{+}|X\right)=f_{S}\left(X_{+}\cap \left(X\times L\right)|X\right)f_{B}\left(X_{+}-\left(X\times L\right)\right), \end{array}$$

where

(12) $$\begin{array}{ccc} f_{S}\left(W|X\right) & = & \Delta \left(W\right)\Delta \left(X\right)1_{L(X)}\left(L\left(W\right)\right){\left[\Phi \left(W;\cdot \right)\right]}^{X}, \end{array}$$

(13) $$\begin{array}{ccc} \Phi (W;x,\ell ) & = & \left\{\begin{array}{cc} p_{S}\left(x,\ell \right)f\left(x_{+}|x,\ell \right), & \mathrm{if}\;\;\;\;(x_{+},\ell )\in W,\\ 1-p_{S}\left(x,\ell \right), & \mathrm{if}\;\;\;\;\ell \notin L(W). \end{array}\right. \end{array}$$

Let $\pi_{k}=\left\{\left(\omega_{k}^{\left(I\right)},p_{k}^{\left(I\right)}\right):I\in F\left(L\right)\right\}$ denote the Mδ-GLMB multi-object posterior density at time *k*. The multi-object prediction density is the Mδ-GLMB

(14) $$\begin{array}{c} \pi_{k+1|k}\left(X\right)=\Delta \left(X\right)\sum_{I\in F(L_{+})}\omega_{k+1|k}^{\left(I\right)}\delta_{I}\left(L\left(X\right)\right){\left[p_{k+1|k}^{\left(I\right)}\right]}^{X}, \end{array}$$

where

(15) $$\begin{array}{ccc} \omega_{k+1|k}^{\left(I\right)} & = & \omega_{S}^{\left(I\right)}\left(I\cap L_{+}\right)\omega_{B}\left(I\cap B_{+}\right), \end{array}$$

(16) $$\begin{array}{ccc} \omega_{S}^{\left(I\right)}\left(L\right) & = & {\left[\eta_{S}^{\left(I\right)}\right]}^{L}\sum_{J⊇L}{\left[1-\eta_{S}^{\left(I\right)}\right]}^{J-L}\omega^{\left(J\right)}, \end{array}$$

(17) $$\begin{array}{ccc} \eta_{S}^{\left(I\right)}\left(\ell \right) & = & \langle p_{S}\left(\cdot ,\ell \right),p^{\left(I\right)}\left(\cdot ,\ell \right)\rangle , \end{array}$$

(18) $$\begin{array}{ccc} p_{k+1|k}^{\left(I\right)}\left(x,\ell \right) & = & 1_{L}+\left(\ell \right)p_{S}^{\left(I\right)}\left(x,\ell \right)+1_{B}+\left(\ell \right)p_{B}\left(x,\ell \right), \end{array}$$

(19) $$\begin{array}{ccc} p_{S}^{\left(I\right)}\left(x,\ell \right) & = & \frac{\langle p_{S}\left(\cdot ,\ell \right)f_{k+1|k}\left(x|\cdot ,\ell \right),p_{k+1|k}^{\left(I\right)}\left(\cdot ,\ell \right)\rangle }{\eta_{S}^{\left(I\right)}\left(\ell \right)}. \end{array}$$

### 3.2. Mδ-GLMB Update

Each state (x,ℓ)∈X has the probability $p_{D}\left(x,\ell \right)$ to be detected, generates a measurement with likelihood function g(z|x,ℓ), and has a probability $1-p_{D}\left(x,\ell \right)$ to be mis-detected. The measurement set *Z* is a superposition of detected objects and Poisson clutter with intensity function κ.

An association map is denoted by θ: L→{0,1,…,|Z|} such that θ(i) = $\theta (i^{\prime })$>0 implies $i=i^{\prime }$. The set Θ of all such association maps is used to denote the association space, and the subset of association maps with domain *I* is denoted by Θ(I).

Conditional on X, detections are independent and clutter is independent of detection, then the multi-object likelihood is given by [28,29]:

(20) $$\begin{array}{c} g\left(Z|X\right)=e^{-\langle \kappa ,1\rangle }\kappa^{Z}\sum_{\theta \in \Theta (L(X))}{\left[\psi_{Z}\left(\cdot ;\theta \right)\right]}^{X}, \end{array}$$

where

(21) $$\begin{array}{c} \psi_{Z}\left(x,\ell ;\theta \right)=\left\{\begin{array}{cc} \frac{p_{D}\left(x,\ell \right)g\left(z_{\theta (\ell )}|x,\ell \right)}{\kappa (z_{\theta (\ell )})}, & \mathrm{if}\;\;\;\;\theta (\ell )>0,\\ 1-p_{D}\left(x,\ell \right), & \mathrm{if}\;\;\;\;\theta (\ell )=0. \end{array}\right. \end{array}$$

Given the Mδ-GLMB multi-object prediction density $\pi_{k+1|k}=\left\{\left(\omega_{k+1|k}^{\left(I\right)},p_{k+1|k}^{\left(I\right)}\right):I\in F\left(L_{+}\right)\right\}$, the Mδ-GLMB updated multi-object density at time k+1 is

(22) $$\begin{array}{c} \pi_{k+1}\left(X|Z\right)=\Delta \left(X\right)\sum_{I\in F(L_{k+1})}\sum_{\begin{array}{c} \theta \in \Theta (I) \end{array}}\omega^{\left(I,\theta \right)}\left(Z\right)\cdot \delta_{I}\left(L\left(X\right)\right){\left[p^{\left(I,\theta \right)}\left(\cdot |Z\right)\right]}^{X}, \end{array}$$

where

(23) $$\begin{array}{ccc} \omega_{k+1}^{\left(I,\theta \right)}\left(Z\right) & \propto & \omega_{k+1|k}^{\left(I\right)}{\left[\eta_{Z}^{\left(I,\theta \right)}\right]}^{I}, \end{array}$$

(24) $$\begin{array}{ccc} \eta_{Z}^{\left(I,\theta \right)}\left(\ell \right) & = & \langle p_{k+1|k}^{\left(\xi \right)}\left(\cdot ,\ell \right),\psi \left(\cdot ,\ell ;\theta \right)\rangle , \end{array}$$

(25) $$\begin{array}{ccc} p_{k+1}^{\left(I,\theta \right)}\left(x,\ell |Z\right) & = & \frac{p_{k+1|k}^{\left(I\right)}\left(x,\ell \right)\psi_{Z}\left(x,\ell ;\theta \right)}{\eta_{Z}^{\left(I,\theta \right)}\left(\ell \right)}. \end{array}$$

The updated Mδ-GLMB density can be denoted by $\pi_{k+1}=\left\{\left(\omega_{k+1}^{\left(I\right)},p_{k+1}^{\left(I\right)}\right):I\in F\left(L_{k+1}\right)\right\}$. Note that the updated density is not an Mδ-GLMB, but a δ-GLMB. Using (8) and (9), a δ-GLMB can be transformed to an Mδ-GLMB.

The ranked assignment and K-shortest paths algorithms are used in [29] to truncate the multi-object posterior and prediction densities, respectively. The algorithm has a cubic complexity in the number of measurements.

Given the posterior Mδ-GLMB density $\pi =\{\left(\omega^{\left(I\right)},p^{\left(I\right)}\right):I\in F\left(L\right)\}$, a tractable suboptimal multi-object estimation can be derived as follows: 

(1) Determine the maximum a posteriori cardinality estimation $N^{*}$ from

(26) $$\begin{array}{ccc} Pr(|X|=n) & = & \sum_{I\in F(L)}\delta_{n}\left(|I|\right)\omega \left(I\right); \end{array}$$

(27) $$\begin{array}{ccc} N^{*} & = & \arg\;\;\max_{n}\;\;Pr\left(|X|=n\right). \end{array}$$

(2) Among the label set with cardinality $N^{*}$, determine the label set $I^{*}$ with highest weight ω(I):

(28) $$\begin{array}{c} I^{*}=\arg\;\;\max_{I}\;\;\omega (I). \end{array}$$

(3) Finally, determine the expected values of the kinematic states $X^{*}$ from $p(x,\ell ;I^{*})$:

(29) $$\begin{array}{c} X^{*}=\left\{\left(x^{*},\ell^{*}\right):\ell^{*}\in I^{*},x^{*}=\arg\;\;\max_{x}\;\;p\left(x,\ell ;I^{*}\right)\right\}. \end{array}$$

## 4. Space Debris Dynamic Model

The tracking algorithm needs a dynamic model of the debris and an observation model to track objects. The dynamic model is represented by the transition density function $f_{k|k-1}\left(X_{k}|X_{k-1}\right)$. It is very difficult to calculate the Markov transition density function of space objects because the space dynamic model is much more complicated than a constant velocity or a constant turn model. However, we can approximate the transition density function of space debris with the help of the software Turboprop and the unscented transform [44]. Turboprop has a package of functions to calculate the trajectories of space objects, and can be called as a function in MATLAB. The elements in Turboprop include: an Earth orientation and atmospheric drag model; the lunar gravity models LP100K, GLGM-2, and LP150Q; the Earth gravity models JGM-3, GGM02C and WGS-84; JPL planetary ephemerides DE403 and DE405; and a solar radiation pressure model.

The Jet Propulsion Laboratory Development Ephemeris (JPL DE) are generally created to support spacecraft missions to the planets. JPL DE designates a series of models consisting of representations of accelerations, velocities, and positions of major Solar System bodies. The acceleration caused by the solar radiation pressure is

(30) $$\begin{array}{c} {\ddot{r}}_{SRP}=p_{SR}c_{R}\frac{A}{m}\frac{\bar{r}}{r}, \end{array}$$

where $p_{SR}$ is the pressure of solar radiation in Pa. $c_{R}$ is the solar radiation coefficient and taken as 1.5 m is the mass of the space debris in kilograms. *A* is the cross-sectional area of the space debris facing the Sun in square meters. *m* and *A* are 0.05 kg and 0.01 m${}^{2}$, respectively. $\bar{r}$ represents the vector from the center of the Sun to the space debris. The mass concentration model is a gravity field, with the total acceleration determined by a series of point masses. This paper takes the Sun, the Earth, Venus, Jupiter, and the Moon into consideration.

The unscented transform is used in the nonlinear projection of mean and covariance estimations. The unscented transform approximates the probability density function (PDF) by a bunch of sigma points. Suppose that each single object density p(x) is a Gaussian mixture of the form $\sum_{i=1}^{N}\omega_{i}N\left(x;m_{i},P_{i}\right)$ and the Gaussian item is *n*-dimensional. Then, the state of space debris is propagated as shown in Table 1.

The sigma-points are chosen as follows:

$$\begin{array}{c} \begin{array}{cccc} \chi^{\left(0\right)}=m, & W^{\left(0\right)}=\frac{\kappa }{n+\kappa }, & j=0 & ,\\ \chi^{\left(j\right)}=m+{\left(\sqrt[{}]{(n+\kappa )P}\right)}_{j}, & W^{\left(j\right)}=\frac{1}{2(n+\kappa )}, & j=1,\ldots ,n & ,\\ \chi^{\left(j\right)}=m-{\left(\sqrt[{}]{(n+\kappa )P}\right)}_{j}, & W^{\left(j\right)}=\frac{1}{2(n+\kappa )}, & j=n+1,\ldots ,2n & , \end{array} \end{array}$$

where *n* represents the dimension of the state vector (6 in this paper), $\sum_{j=0}^{2n}W^{\left(j\right)}=1$, ${\left(\sqrt[{}]{(n+\kappa )P}\right)}_{j}$ is the *j*th row or column of the square root $\sqrt[{}]{(n+\kappa )P}$, κ is the scaling parameter (2 in this paper).

## 5. Estimation of Objects Outside the Observation Volume

Most of the literature in object tracking assumes that objects do not go outside of the observation volume. This is not the case for space debris tracking. Filters with no special consideration of detection probability setting cannot provide estimation for objects outside the observation volume because there are no measurements available. However, since the observation model only affects the update and not the prediction, the estimation of objects can still be provided because the prediction is always available. Aiming to provide estimation for the whole area, we set the detection probability to zero for areas outside of the observation volume.

We assume that the single object density is represented by a Gaussian mixture (GM). Since UKF is used in this paper for the space debris transition function, each Gaussian item is represented by a bunch of sigma-points. If all sigma-points are inside or outside the observation volume, the detection probability for this Gaussian item is set to $p_{Din}$ and $p_{Dout}$, respectively. $p_{Din}$ is the detection probability inside the observation volume and $p_{Dout}$ is the detection probability outside the observation volume. Otherwise, the EM algorithm is used to build a new GM to approximate the current Gaussian item.

**Figure 1:**
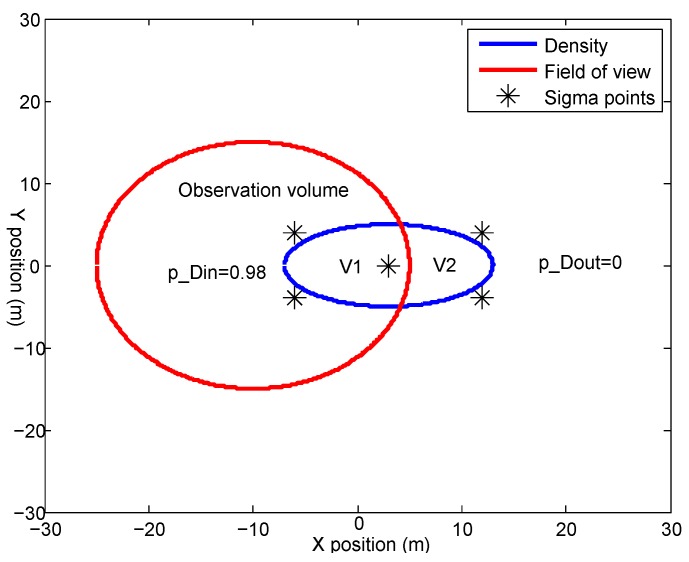
The predicted probability density function is shown by a Gaussian mixture (GM). The blue circle is one Gaussian item. V1 represents the part inside the observation volume, and V2 represents the outside part.

An illustration is shown in Figure 1.

The predicted probability density function is shown by a GM. The blue circle is one Gaussian item. V1 represents the part inside the observation volume, and V2 represents the outside part. ∗ represents the sigma points obtained by the unscented transform. $p_{Din}=0.98$ is an example of the detection probability inside the observation volume. Since there is no measurement from outside the observation volume, the detection probability is set to $p_{Dout}=0$. The EM algorithm is used to build a new GM to represent V1 and V2, respectively.

The EM algorithm is an efficient method to find the maximum-likelihood estimate of the unknown parameters of an underlying distribution from a given data set. Usually, the data set has missing values or is incomplete. Each iteration of the EM algorithm has two procedures: The expectation step (E-step) and the maximization step (M-step). The E-step creates a function for the expectation of the log-likelihood evaluated using the current estimate for the parameters. In the M-step, the likelihood function is maximized under the assumption that the missing data are known.

If the sigma-points of one Gaussian item are on both sides of the observation edge, then N=1000 points are sampled from this Gaussian distribution. Our experience shows that 1000 points are enough to represent the probability distribution in the experiment. Depending on whether the points are inside the observation volume or not, these points are divided into two groups: $Points_{in}$ and $Points_{out}$. $Points_{in}$ are the sampled points inside the observation volume and $Points_{out}$ are the sampled points outside the observation volume. The EM algorithm is used afterwards for a Gaussian mixture parameter estimation. Each Gaussian item is represented by two new Gaussian mixtures. That is,

(31) $$\begin{array}{c} \begin{array}{c} \sum_{i=1}^{J^{\left(\xi \right)}\left(\ell \right)}\omega_{i}^{\left(\xi \right)}\left(\ell \right)N\left(x;m_{i}^{\left(\xi \right)}\left(\ell \right),P_{i}^{\left(\xi \right)}\left(\ell \right)\right)=\\ \sum_{i=1}^{J^{\left(\xi \right)}\left(\ell \right)}\omega_{i}^{\left(\xi \right)}\left(\ell \right)\left(\sum_{h=1}^{H^{\left(\xi \right)}\left(\ell \right)}\omega_{h}^{\left(\xi \right)}\left(\ell \right)N\left(x;m_{h}^{\left(\xi \right)}\left(\ell \right),P_{h}^{\left(\xi \right)}\left(\ell \right)\right)+\sum_{k=1}^{K^{\left(\xi \right)}\left(\ell \right)}\omega_{k}^{\left(\xi \right)}\left(\ell \right)\left(x;m_{k}^{\left(\xi \right)}\left(\ell \right),P_{k}^{\left(\xi \right)}\left(\ell \right)\right)\right). \end{array} \end{array}$$

**Figure 2:**
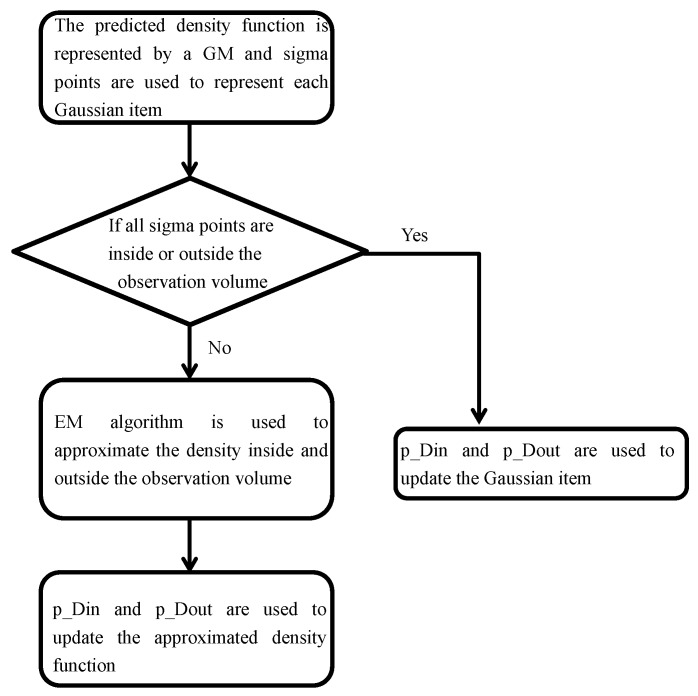
Estimation for objection outside the observation volume. EM: expectation maximization.

The first GM is to represent V1 and the second GM is to represent V2. An illustration of the method is shown in Figure 2.

The estimation of objects is available when they are outside the observation volume. The proposed consensus Mδ-GLMB can be used for sensors with different observation volumes.

The parameters in the density approximation are the number of Gaussian items, the weights for the items, the covariance matrices, and the means. These parameters are chosen as follows.

(1) The number of Gaussian components:

The number of new Gaussian mixture components is important in the density approximation with the EM algorithm. However, it is very difficult to derive the number of Gaussian components. In practice, we choose a reasonably large number ($J_{max}=10$ in this paper) as the maximum number of new Gaussian mixture items in the approximation. The EM algorithm is used for situations with a different number of Gaussian components $\left(J_{min}=1,\ldots ,J_{max}=10\right)$. Then, the Bayesian information criterion (BIC) is used to determine the best number of Gaussian components. The BIC is a criterion for model selection among a finite set of models [45]. The model with the lowest BIC is preferred. The BIC is defined as follows:

(32) $$\begin{array}{c} BIC(J,\theta ,P)=-2log\;\;L(\theta ,J|P)+M\;ln(N), \end{array}$$

where *J* is the number Gaussian items set for the approximation, θ is the estimated parameter values that maximize the likelihood function, *P* is the sampled data, L(·) is the maximized value of the likelihood function, *M* is the number of parameters estimated (6 in this paper), and *N* is the number of sampled points (1000 in this paper).

(2) The weights for the Gaussian items:

The weights can be the same and set as $W_{j}=\frac{1}{J}$.

(3) The covariance and the means:

The means of the Gaussian position components can be set by uniformly sampling in the observation volume. The means of the Gaussian velocity components can be set to zero. The initial covariance is chosen by experience, which is the the same covariance matrix of the Gaussian item that was sampled from.

## 6. Distributed Information Fusion with Consensus for Mδ-GLMB

After each node finishes the state estimation of space debris, a distributed information fusion can be performed to achieve better results. The consensus algorithm is used in this paper to achieve distributed information fusion. This section shows that the Mδ-GLMB is algebraically closed under Kullback–Leibler averaging (KLA) (i.e., the KLA of Mδ-GLMB is also Mδ-GLMB) [18]. The closed form expression for KLA of the Mδ-GLMB is derived and used for consensus fusion of the Mδ-GLMB posterior density [18].

### 6.1. Consensus for Mδ-GLMB Filtering

The assumption that the estimation from each node in a multi-sensor network is independent or that the correlations among them are known is wrong. The correlation is commonly caused by data incest. Data incest happens when the same information takes several paths from the other sensors to the fusion center and the raw measurements are inadvertently used multiple times. Centralized information fusion is carried out by the Bayes solution:

(33) $$\begin{array}{c} f\left(X|Z_{a}\cup Z_{b}\right)=\frac{f\left(Z_{a}\cup Z_{b}|X\right)f\left(X\right)}{f(Z_{a}\cup Z_{b})}. \end{array}$$

The result can be carried out as follows if the information is treated as being independent:

(34) $$\begin{array}{c} f\left(X|Z_{a}\cup Z_{b}\right)=\frac{f\left(Z_{a}|X\right)f\left(Z_{b}|X\right)f\left(X\right)}{f\left(Z_{a}\right)f\left(Z_{b}\right)}. \end{array}$$

Since there is a common measurement history and common process noise, the information from each node are not independent from each other. Data incest effects can be effectively counteracted by the consensus algorithm.

Given labeled multi-object densities $\pi^{\left(i\right)}$ on F(X×L),i∈I, and the normalized non-negative weights $w^{\left(i\right)},i\in I$, the weighted KLA is defined by

(35) $$\begin{array}{c} \bar{\pi }≜\arg\;\min_{\pi }\sum_{i\in I}w^{\left(i\right)}D_{KL}\left(\pi |\right|\pi^{\left(i\right)}), \end{array}$$

where

(36) $$\begin{array}{c} D_{KL}\left(\pi |\right|\pi^{\left(i\right)})≜\int \pi \left(X\right)\log\left(\frac{\pi (X)}{\pi^{\left(i\right)}\left(X\right)}\right)\delta X \end{array}$$

is the Kullback–Leibler Divergence (KLD) of $\pi^{\left(i\right)}$ from π [20,22]. The set integral is used in the integral for any function *f* on F(X×L):

(37) $$\begin{array}{c} \int f\left(X\right)\delta X=\sum_{i=0}^{\infty }\frac{1}{i!}\sum_{\left(\ell_{1},\ldots ,\ell_{i}\right)\in L^{i}}\int f\left(\left\{\left(x_{1},\ell_{1}\right),\ldots ,\left(x_{i},\ell_{i}\right)\right\}\right)d\left(x_{1},\ldots ,x_{i}\right). \end{array}$$

Given normalized non-negative weights $w^{\left(i\right)},i\in I$ and labeled multi-object densities $\pi^{\left(i\right)},i\in I$, then the weighted KLA in (35) is

(38) $$\begin{array}{c} \bar{\pi }=\underset{i\in I}{⊕}\left(w^{\left(i\right)}⊙\pi^{\left(i\right)}\right), \end{array}$$

where

(39) $$\begin{array}{c} \underset{i\in I}{⊕}\left(w^{\left(i\right)}⊙\pi^{\left(i\right)}\right)≜\frac{\prod_{i\in I}{\left[\pi^{\left(i\right)}\left(X\right)\right]}^{w^{\left(i\right)}}}{\int \prod_{i\in I}{\left[\pi^{\left(i\right)}\left(X\right)\right]}^{w^{\left(i\right)}}\delta X} \end{array}$$

is the normalized weighted geometric mean [18]. It was shown in [13] that independent, identically distributed cluster and Poisson RFSs are algebraically closed under KL averaging. The Mδ-GLMB family is also algebraically closed under KL averaging.

The following result shows that the KLA of the Mδ-GLMB densities is also a Mδ-GLMB density [18].

Given the Mδ-GLMB densities $\pi^{\left(i\right)}=\left\{\left(\omega_{i}^{\left(I\right)},p_{i}^{\left(I\right)}\right):I\in F\left(L\right)\right\},i\in I$ and normalized non-negative weights $w^{\left(i\right)},i\in I$, the KLA, and hence the normalized weighted geometric mean, is an Mδ-GLMB given by [18]

(40) $$\begin{array}{c} \bar{\pi }=\underset{i\in I}{⊕}\left(w^{\left(i\right)}⊙\pi^{\left(i\right)}\right)=\left\{\left({\bar{\omega }}^{\left(L\right)},{\bar{p}}^{\left(L\right)}\right):L\in F\left(L\right)\right\}, \end{array}$$

where

(41) $$\begin{array}{ccc} {\bar{\omega }}^{\left(L\right)} & = & \frac{\prod_{i\in I}{\left(\omega_{i}^{\left(L\right)}\right)}^{w^{\left(i\right)}}{\left[\int \prod_{i\in I}{\left(p_{i}^{\left(L\right)}\left(x,\cdot \right)\right)}^{w^{\left(i\right)}}\right]}^{L}}{\sum_{J\subseteq L}\prod_{i\in I}{\left(\omega_{i}^{\left(J\right)}\right)}^{w^{\left(i\right)}}{\left[\int \prod_{i\in I}{\left(p_{i}^{\left(J\right)}\left(x,\cdot \right)\right)}^{w^{\left(i\right)}}\right]}^{J}}, \end{array}$$

(42) $$\begin{array}{ccc} {\bar{p}}^{\left(L\right)}\left(\cdot ,\ell \right) & = & \frac{\prod_{i\in I}{\left(p_{i}^{\left(L\right)}\left(\cdot ,\ell \right)\right)}^{w^{\left(i\right)}}}{\int \prod_{i\in I}{\left(p_{i}^{\left(L\right)}\left(x,\ell \right)\right)}^{w^{\left(i\right)}}dx}. \end{array}$$

The component $\left({\bar{\omega }}^{\left(L\right)},{\bar{p}}^{\left(L\right)}\left(\cdot \right)\right)$ of the KLA Mδ-GLMB can be rewritten as

(43) $$\begin{array}{ccc} {\bar{\omega }}^{\left(L\right)} & \propto & \prod_{i\in I}{\left(\omega_{i}^{\left(L\right)}\right)}^{w^{\left(i\right)}}{\left[\int \prod_{i\in I}{\left(p_{i}^{\left(L\right)}\left(x,\cdot \right)\right)}^{w^{\left(i\right)}}\right]}^{L}, \end{array}$$

(44) $$\begin{array}{ccc} {\bar{p}}^{\left(L\right)}\left(\cdot \right) & = & \underset{i\in I}{⊕}\left(w^{\left(i\right)}⊙p_{i}^{\left(L\right)}\left(\cdot \right)\right), \end{array}$$

where (44) is the Chernoff fusion rule for the single-object PDFs. It can be seen from (43) and (44) that each fused Mδ-GLMB component $\left({\bar{\omega }}^{\left(L\right)},{\bar{p}}^{\left(L\right)}\left(\cdot \right)\right)$ can be independently calculated, which makes the fusion procedure fully parallelizable.

Given a node network N with multi-object probability densities $\pi^{\left(i\right)}$ from each node *i*, and normalized non-negative weights $w^{\left(i,j\right)}$ for node *i* to nodes $j\in N^{\left(i\right)}$, with $\sum_{j\in N^{\left(i\right)}}w^{\left(i,j\right)}=1$, the KLA over the whole network can be calculated in a scalable and distributed way using the consensus algorithm. Suppose that each node starts with initial PDF $\pi_{0}^{\left(i\right)}=\pi$. The *n*th consensus can be calculated by

(45) $$\begin{array}{c} \pi_{n}^{\left(i\right)}=\underset{j\in N}{⊕}\left(w_{n}^{\left(i,j\right)}⊙\pi^{\left(j\right)}\right), \end{array}$$

where $w_{n}^{\left(i,j\right)}$ is the (i,j)-th entry of the matrix $\Omega^{n}$. The (i,j)-th entry of the consensus matrix Ω is given by $w^{\left(i,j\right)}1_{N^{\left(i\right)}}\left(j\right)$. The consensus algorithm enjoys some good convergence properties. It was shown that if the consensus matrix is doubly stochastic (all rows and columns sum up to 1) and primitive (there exists an integer *m* that $\Omega^{m}$ has all positive entries), then

(46) $$\begin{array}{c} \lim_{n\to \infty }w_{n}^{\left(i,j\right)}=\frac{1}{\left|N\right|}. \end{array}$$

So, with a doubly stochastic and primitive consensus matrix, the global unweighted KLA of the densities over the whole network can be calculated by the consensus iterative of each node as the number of consensus steps tends to infinity [13].

A necessary condition for the consensus matrix to be primitive is that the network is strongly connected, which means that for any pair of nodes (i,j)∈N there exists a directed path from node *i* to node *j* and vice versa. This condition can also be satisfied if $w^{\left(i,j\right)}>0\;\forall i\in N,j\in N^{\left(i\right)}$. The consensus matrix is primitive and doubly stochastic for a undirected network (i.e., whenever node *i* sends information to node *j*, it also receives information from node *j*) if [46]:

(47) $$\begin{array}{c} w^{\left(i,j\right)}=\left\{\begin{array}{cc} \frac{1}{1+\max\{|N^{\left(i\right)}|,|N^{\left(j\right)}|\}}, & i\in N,j\in N^{\left(i\right)}\backslash \left\{i\right\},\\ 1-\sum_{j\in N^{\left(i\right)}\backslash \left\{i\right\}}w^{\left(i,j\right)}, & i\in N,j=i. \end{array}\right. \end{array}$$

The global unweighted KLA of the multi-object posterior densities can be achieved by iterative local KLA averaging as the consensus step tends to infinity. In practice, the iteration is stopped at some finite number.

A Gaussian mixture (GM) is a typical choice to represent single-object densities. The fusion rule involves multiplication and exponentiation of GMs which generally do not provide a GM. A suitable approximation of the GM exponentiation has to be devised in order to preserve the GM form. For the sake of simplicity, let us consider only two agents (labeled *a* and *b*) with Gaussian mixture location densities

(48) $$\begin{array}{c} p^{i}\left(x\right)=\sum_{j=1}^{N_{G}^{i}}\alpha_{j}^{i}N\left(x;{\bar{x}}_{j}^{i},P_{j}^{i}\right),i=a,b. \end{array}$$

The fused location PDF is also a Gaussian mixture, and can be approximated as follows:

(49) $$\begin{array}{c} \bar{p}\left(x\right)=\frac{\sum_{i=1}^{N_{G}^{a}}\sum_{j=1}^{N_{G}^{b}}\alpha_{ij}^{ab}N\left(x;{\bar{x}}_{ij}^{ab},P_{ij}^{ab}\right)}{\sum_{i=1}^{N_{G}^{a}}\sum_{j=1}^{N_{G}^{b}}\alpha_{ij}^{ab}}, \end{array}$$

where

(50) $$\begin{array}{ccc} P_{ij}^{ab} & = & {\left[\omega {\left(P_{i}^{a}\right)}^{-1}+\left(1-\omega \right){\left(P_{j}^{b}\right)}^{-1}\right]}^{-1}, \end{array}$$

(51) $$\begin{array}{ccc} {\bar{x}}_{ij}^{ab} & = & P_{ij}^{ab}\left[\omega {\left(P_{i}^{a}\right)}^{-1}{\bar{x}}_{i}^{a}+\left(1-\omega \right){\left(P_{j}^{b}\right)}^{-1}{\bar{x}}_{j}^{b}\right],\\ \alpha_{ij}^{ab} & = & {\left(\alpha_{i}^{a}\right)}^{\omega }{\left(\alpha_{j}^{b}\right)}^{1-\omega }\kappa \left(\omega ,P_{i}^{a}\right)\kappa \left(1-\omega ,P_{j}^{b}\right), \end{array}$$

(52) $$\begin{array}{c} \cdot N({\bar{x}}_{i}^{a}-{\bar{x}}_{j}^{b};0,\frac{P_{i}^{a}}{\omega }+\frac{P_{j}^{b}}{1-\omega }), \end{array}$$

(53) $$\begin{array}{ccc} \kappa (\omega ,P) & = & \frac{{\left[det\left(2\pi P\omega^{-1}\right)\right]}^{\frac{1}{2}}}{{\left[det\left(2\pi P\right)\right]}^{\frac{\omega }{2}}}. \end{array}$$

The validity of the equations above depends on the cross-products of the different terms in the Gaussian mixture being negligible, which requires that the Gaussian components are well separated. This condition is well-met since a suitable merging step is used to fuse Gaussian components with Mahalanobis distance [47] below a given threshold. The fusion can be easily extended to more nodes by sequentially applying the pairwise fusion (50) and (51).

The other common way to represent a single-object density is by particles. The local filtering steps are more resource-demanding than a GM implementation. Moreover, the in-node computation burden is increased compared to GM representation because the information fusion requires additional techniques (e.g., least square estimation, kernel density estimation, or parametric model approaches).

### 6.2. Discussions on Consensus

**Figure 3:**
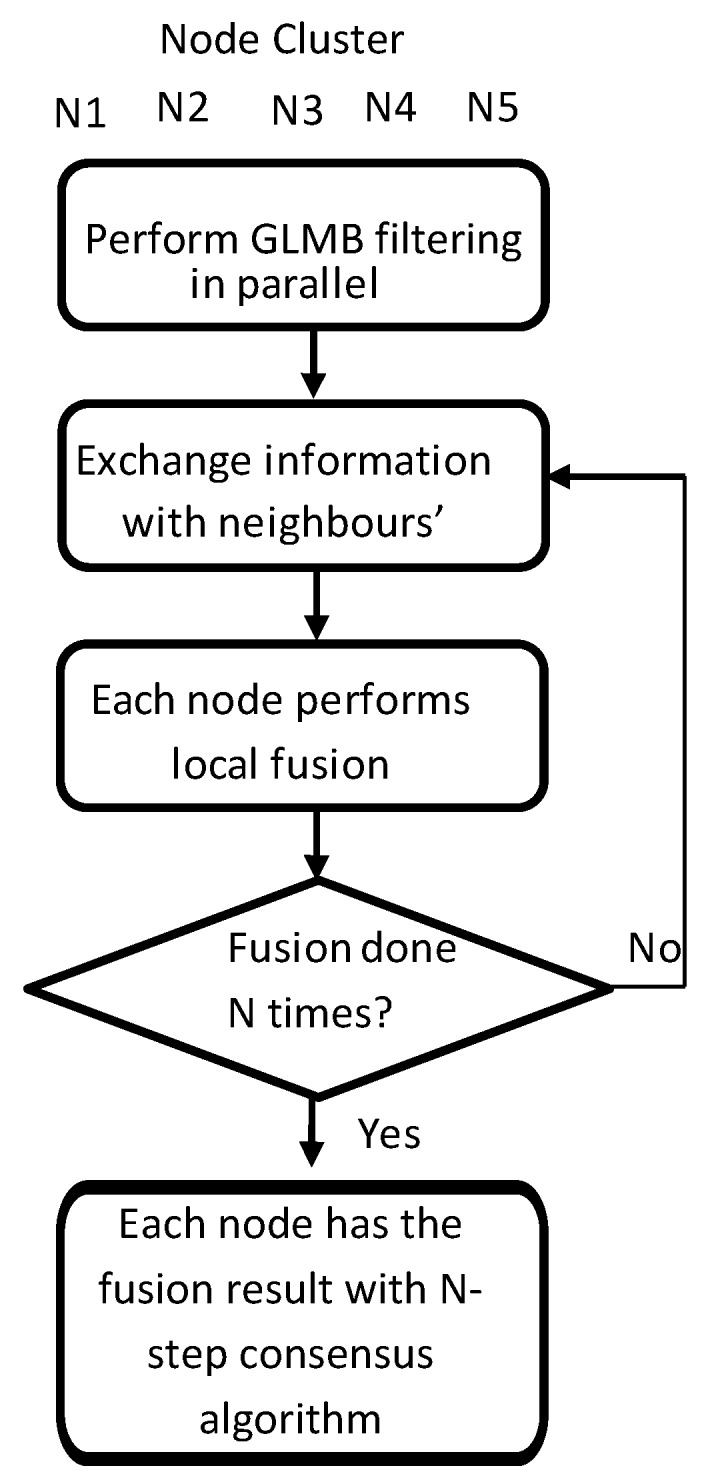
System design.

Each node in the network performs local prediction and update. They then exchange information with their neighbors. Information fusion with the consensus algorithm is carried out afterwards. Depending on the requirements of the system, the procedure can be repeated N times, which is called N-steps of consensus. An illustration of the system design is shown in Figure 3.

The network used in this paper is a fully distributed one. Every node performs the same operations: updates its own information, exchanges information with its neighbors, and performs information fusion using the consensus algorithm.

The consensus algorithm achieves global averaging over the whole network with iterative averaging among neighboring nodes for each node. The main disadvantage is the computation complexity. With an increase of network size, the computation load for the whole network and the time it takes to reach global averaging significantly increases. The performance degradation is mainly due to the time it takes for the information from each node to be transferred to the rest of the nodes. In practice, the number of nodes in the whole network is chosen according to the computation power of each node so that the time required for the consensus algorithm is kept under a certain threshold.

It was proved in [48] that the complexity of the centralized Mδ-GLMB filter is linearly related to the number of sensors. Each node in the consensus Mδ-GLMB filtering system has to carry out local prediction and local update. The computation complexity is independent of the number of nodes. The computation complexity of the consensus algorithm for each node only depends on the number of neighboring nodes, and has nothing to do with the total number of nodes in the whole network.

As the number of targets increases, tracking becomes more challenging. Even though the ranked assignment algorithm and K-shortest paths algorithm are used to truncate the posterior densities and prediction densities, respectively, the filtering still has cubic complexity in the number of measurements. An efficient implementation of the GLMB filter by combining the prediction and update into a single step was proposed in [30]. The earlier implementation involves separate truncations in the prediction and update steps. The proposed implementation requires only one truncation procedure for each iteration. Furthermore, an efficient algorithm based on Gibbs sampling for GLMB filtering density truncation was also proposed in [30]. The implementation has a linear complexity in the number of measurements and a quadratic one in the number of objects. The Mδ-GLMB filter used in this paper was proposed in [31]. It is a tractable multi-object density approximation that can capture statistical dependence between objects. It matches the cardinality distribution and the first moment of the labeled multi-object distribution of interest. The performance of Mδ-GLMB filtering is sufficient for the situation in this paper.

The computation load of fusion for each node only depends on the consensus steps and the number of neighbors. For example, if the diameter of a network is 3, then it takes three steps at most for information in any node to be transferred to any other node in this network. As the number of nodes increases, it takes more time to reach the global average. However, the computation for each node at one time is the same, and only depends on the number of neighbors. Large networks will only be limited by the computation capability of each node. Therefore, the system is scalable and the processing load for each node is scalable with respect to the size of the network.

Fully decentralized systems suffer from the problem of synchronization and inconsistency. As the number of nodes increases, it becomes more difficult to achieve synchronization in the system. The effect of communication delays becomes more severe in larger networks. Even if the time delays are small, they can deteriorate the system’s performance or even destabilize it. The output synchronization of nonlinear systems with communication time delays was discussed in [49]. A new framework was proposed in [50] to address the consensus with multi-agent systems and the synchronization of complex networks. Coordination can be achieved for the discrete-time delayed systems with linear dynamics [51] and switching topologies [52].

A space debris tracking approach with GM-CPHD and consensus algorithm was presented in [42]. The performance of the approach was shown with an example of 45 clusters of sensors to achieve global tracking of space debris. While the performance was very good, the approach used in this paper has much better results. This is because the Mδ-GLMB filter has a more accurate propagation of the posterior density than the CPHD filter, which makes the Mδ-GLMB filter better able to locate targets. Another disadvantage of the CPHD filter is the “spooky” effect which causes the CPHD filter to temporarily drop tracks which are subjected to missed detections and to declare multiple estimates for existing tracks in place of the dropped tracks [29]. Further, the CPHD filter cannot provide the trajectories of the targets. The estimates of the target from the CPHD filter are indistinguishable. The Mδ-GLMB filter used in this paper significantly outperforms the CPHD filter used in [42].

This paper presents a scalable solution to the problem of tracking space debris. The space tracking approach employed in this paper is an improvement over existing methods in several aspects. Firstly, the core elements of data association, detection, and tracking are solved with an integrated approach based on labeled RFS in this paper, while most SSA literature treats them separately. Secondly, most SSA methods use a single sensor (e.g., [4,35,41,53]) or a centralized tracking system as in [6] to track space objects. The consensus algorithm used in this paper has better performance than single-sensor methods and has a smaller computation load than a centralized tracking system. Thirdly, the consensus approach used in this paper can provide estimation for targets outside of the observation volume and can be used for information fusion for sensors with different observation volumes, unlike most consensus algorithms (e.g., [13,18,54]) or other distributed information fusion algorithms (e.g., [55,56]) in the current literature which can only be used for sensors with the same observation volume.

The consensus iterate of each node converges to the global unweighted KLA of the multi-object posterior densities as *n* tends to infinity. In practice, consensus steps N is chosen based on the network size. A methodology to achieve distributed average consensus in finite time was proposed in [57]. The proposed algorithm requires less memory at the cost of a slight increase in the number of steps required for termination. To relax the assumption that the nodes are aware of the upper bound on the network diameter, the authors provide an upper bound on the network diameter which, in the worst case, is twice the actual diameter. Ref. [58] reaches consensus in finite time using only linear iterations. The authors show that finite-time average consensus can always be achieved for undirected networks.

This paper treats the consensus step *N* as prior information. *N* is broadcasted to every node before the information fusion. This is certainly possible, but it becomes difficult when the size of the network grows. There are some approaches that do not require continuous data exchange nor knowledge on a global parameter. Ref. [59] reviews some of the main discrete and finite time average consensus implementations, from theoretical and empirical points of view. Three main aspects, namely *computational analysis*, *packet loss resilience analysis*, and *stealth attacks resilience analysis*, are analyzed. The authors of [59] first review synchronous and uniformly sampled approaches which are based on the standard average consensus iteration [60], on the flooding approach [57], or on a repetition of the max-consensus procedure [57]. They then present event-based finite-time average consensus implementations with point-to-point communication capability based on the Converge Cast [61] and on the token-passing approaches [62].

## 7. Numerical Results

The goal of this paper is to show the efficacy of consensus Mδ-GLMB in tracking space debris. We demonstrate this through three experiments. The first experiment was designed to show that the Mδ-GLMB filter proposed in this paper with special consideration of detection probability setting can provide estimation for objects outside the observation volume. The second experiment was designed to show that consensus fusion can be performed among sensors with different observation volumes. The third experiment was designed to show that the consensus algorithm can significantly improve the tracking performance when all objects are inside the observation volumes of all sensors.

The object state is a vector of position and velocity, $x_{k}={\left[p_{x}\;\;p_{y}\;\;p_{z}\;\;v_{x}\;\;v_{y}\;\;v_{z}\right]}^{T}$. The state dynamic model is:

$$\begin{array}{c} x_{k+1}=f\left(x_{k}\right)+w_{k}. \end{array}$$

The first step is to build the space debris trajectories with the help of Turboprop. f(·) is the transition density function and $w_{k}∼N\left(\cdot ;0,Q\right)$ is the process noise with $Q=diag(\left[\sigma_{px}^{2},\sigma_{py}^{2},\sigma_{pz}^{2},\sigma_{vx}^{2},\sigma_{vy}^{2},\sigma_{vz}^{2}\right])$, $\sigma_{px}$ = $\sigma_{py}$ = $\sigma_{pz}$ = 1 km, $\sigma_{vx}$ = $\sigma_{vy}$ = $\sigma_{vz}$ = 0.01 km/s. The birth model to generate the simulation scenario is a labeled multi-Bernoulli RFS with parameters $\pi_{B}={\left\{r_{B}^{\left(i\right)},p_{B}^{\left(i\right)}\right\}}_{i=1}^{5}$, where $r_{B}^{\left(i\right)}=0.02$ and $p_{B}^{\left(i\right)}\left(x\right)=N\left(x;m_{B}^{\left(i\right)},P_{B}\right)$, with

$$\begin{array}{c} \begin{array}{cc} m_{\gamma }^{\left(1\right)}= & {\left[42,097.71\;\mathrm{km},-2221.31\;\mathrm{km},-3.68\;\mathrm{km},0.1620\;\mathrm{km}/s,3.0703\;\mathrm{km}/s,6.7817\;\mathrm{km}/s\right]}^{T},\\ m_{\gamma }^{\left(2\right)}= & {\left[42,097.31\;\mathrm{km},-2097.2\;\mathrm{km},-756.51\;\mathrm{km},0.1625\;\mathrm{km}/s,2.8851\;\mathrm{km}/s,1.0501\;\mathrm{km}/s\right]}^{T},\\ m_{\gamma }^{\left(3\right)}= & {\left[42,096.91\;\mathrm{km},-1719.39\;\mathrm{km},-1425.14\;\mathrm{km},0.1628\;\mathrm{km}/s,2.3520\;\mathrm{km}/s,1.9735\;\mathrm{km}/s\right]}^{T},\\ m_{\gamma }^{\left(4\right)}= & {\left[42,098.08\;\mathrm{km},-2083.03\;\mathrm{km},745.34\;\mathrm{km},0.1613\;\mathrm{km}/s,2.8852\;\mathrm{km}/s,-1.0500\;\mathrm{km}/s\right]}^{T},\\ m_{\gamma }^{\left(5\right)}= & {\left[42,098.43\;\mathrm{km},-1700.09\;\mathrm{km},1407.22\;\mathrm{km},0.1610\;\mathrm{km}/s,2.3522\;\mathrm{km}/s,-1.9734\;\mathrm{km}/s\right]}^{T},\\ P_{\gamma }= & diag([10\;\mathrm{km},10\;\mathrm{km},10\;\mathrm{km},0.01\;\mathrm{km}/s,0.01\;\mathrm{km}/s,0.01\;\mathrm{km}/s]). \end{array} \end{array}$$

$r_{B}^{\left(i\right)}$ is the birth intensity, which is the expected number of new objects born at each time step, at each object birth place *i*. All targets are initially born inside the observation volume of a sensor (not necessarily the same one). The situation where a target is born outside of the observation volume of all sensors and goes from outside to inside of the observation volume can be handled by a measurement-based birth model, which is beyond the scope of this paper. The space object trajectories generated by the birth model in this experiment are very common in real space object tracking scenarios.

Clutter is modeled as a Poisson RFS with intensity $\kappa_{k}\left(z\right)=\lambda_{c}Vu\left(z\right)$. $\lambda_{c}$ is the average of clutter returns per unit volume, which was 100 in the simulation. u(·) is the uniform density over the observation region, *V* is the “volume” of the observation region.

All nodes used in this paper were located on the surface of the Earth and rotated with the Earth. The latitudes and longitudes of nodes were

$$\begin{array}{c} L1=\left(0^{\circ },0^{\circ }\right),L2=\left(0^{\circ },30^{\circ }N\right),L3=\left(0^{\circ },30^{\circ }S\right),L4=\left(30^{\circ }E,0^{\circ }\right),L5=\left(30^{\circ }W,0^{\circ }\right), \end{array}$$

respectively. Node1 was initially on the *x*–*z* plane.

The sensor’s observation is a noisy bearing and range vector given by

(54) $$\begin{array}{c} z_{k}=\left[\begin{array}{c} \sqrt{p_{x,k}^{2}+p_{y,k}^{2}+p_{z,k}^{2}}\\ \arctan\left(\frac{p_{y,k}}{p_{x,k}}\right)\\ \arctan\left(\frac{p_{z,k}}{\sqrt{p_{x,k}^{2}+p_{y,k}^{2}}}\right) \end{array}\right]+\varepsilon_{k}, \end{array}$$

where $\varepsilon_{k}∼N\left(\cdot ;0,R_{k}\right)$ with

$$\begin{array}{c} R_{k1}=diag\left(\left[\sigma_{r1}^{2},\sigma_{\alpha 1}^{2},\sigma_{\beta 1}^{2}\right]\right),\sigma_{r1}=0.05\;\;\mathrm{km},\sigma_{\alpha 1}=0.02^{\circ }\;\;\sigma_{\beta 1}=0.02^{\circ },\;\;\\ R_{k2}=diag\left(\left[\sigma_{r2}^{2},\sigma_{\alpha 2}^{2},\sigma_{\beta 2}^{2}\right]\right),\sigma_{r2}=0.22\;\;\mathrm{km},\sigma_{\alpha 2}=0.01^{\circ }\;\;\sigma_{\beta 2}=0.01^{\circ },\;\;\\ R_{k3}=diag\left(\left[\sigma_{r3}^{2},\sigma_{\alpha 3}^{2},\sigma_{\beta 3}^{2}\right]\right),\sigma_{r3}=0.12\;\;\mathrm{km},\sigma_{\alpha 3}=0.02^{\circ }\;\;\sigma_{\beta 3}=0.01^{\circ },\;\;\\ R_{k4}=diag\left(\left[\sigma_{r4}^{2},\sigma_{\alpha 4}^{2},\sigma_{\beta 4}^{2}\right]\right),\sigma_{r4}=0.1\;\;\mathrm{km},\sigma_{\alpha 4}=0.01^{\circ }\;\;\sigma_{\beta 4}=0.02^{\circ },\;\;\\ R_{k5}=diag\left(\left[\sigma_{r5}^{2},\sigma_{\alpha 5}^{2},\sigma_{\beta 5}^{2}\right]\right),\sigma_{r5}=0.15\;\;\mathrm{km},\sigma_{\alpha 5}=0.015^{\circ }\;\;\sigma_{\beta 5}=0.015^{\circ }. \end{array}$$

Δ = 40 s was the sampling period. The detection probability inside the observation volume $p_{Din}=0.98$. The δ-GLMB filter was capped to 10,000 components.

In order to show statistical significance, results were shown over 100 Monte Carlo trials for the same object trajectories but different, independently generated, clutter and measurement noise realizations. The first experiment showed the efficacy of the algorithm with one realization. The second and third experiments showed with the mean value over 1000 Monte Carlo trials. Typically, the optimal sub-pattern assignment (OSPA) performance in the RFS area is shown with mean values. The OSPA distance is the sum of two parts: location error and cardinality error. The cardinality error is shown with not just the mean value, but also the standard deviation.

Performance evaluation of the multi-target algorithm is of great practical importance in the design and comparison of tracking systems. The difference between the estimation and true trajectories is very small compared with the motion range of space debris. Therefore, the OSPA metric was used to evaluate the performance with Euclidean distance *p* = 2, and cutoff parameter *c* = 20 km for the second experiment and *c* = 1 km for the third experiment. The OSPA is a mathematically and intuitively consistent metric for multi-object filtering performance evaluation [63]. OSPA distance has two components: localization and cardinality. The total OSPA distance is the sum of the two components. The cut-off parameter *c* determines the penalty weighting for cardinality errors as opposed to localization errors. The second experiment was mainly designed to show the estimation performance for targets when they go outside the observation volume. As there were no measurements available, the estimation error became larger and larger, and so did the OSPA value. Therefore, *c* = 20 km was used in this experiment. The third experiment was mainly intended to show the efficacy of the consensus algorithm when targets were all inside the combined observation volumes. Since measurements were available all the time, the estimation error was smaller than in the second experiment. So, the cut-off value *c* = 2 km was used in the third experiment.

The consensus algorithm stops at N steps. The information of N should be broadcasted to every node beforehand and treated as prior information. In practice, N is chosen based on the size of the network. This paper shows the estimation performance with 1-step consensus, 2-step consensus, and 3-step consensus.

### 7.1. Estimation of Objects Outside the Observation Volume

This section demonstrates that estimation can be provided for objects outside the observation volume by setting the detection probability outside the observation volume to zero. Only node1 is used in this section. A fixed number of objects are presented here for simplicity. All objects are inside the observation volume initially, but as time goes on, some objects go outside. The field of view for node1 is

$$\begin{array}{c} V1=\left[30,000\;\mathrm{km}\;\;\;40,000\;\mathrm{km}\right]\;\times \left[-15^{\circ }\;\;0^{\circ }\right]\;\times \left[-5^{\circ }\;\;5^{\circ }\right]. \end{array}$$

This yields a sensor volume of 150 $\deg^{2}$. Some objects go outside of the observation volume with this setting.

**Figure 4:**
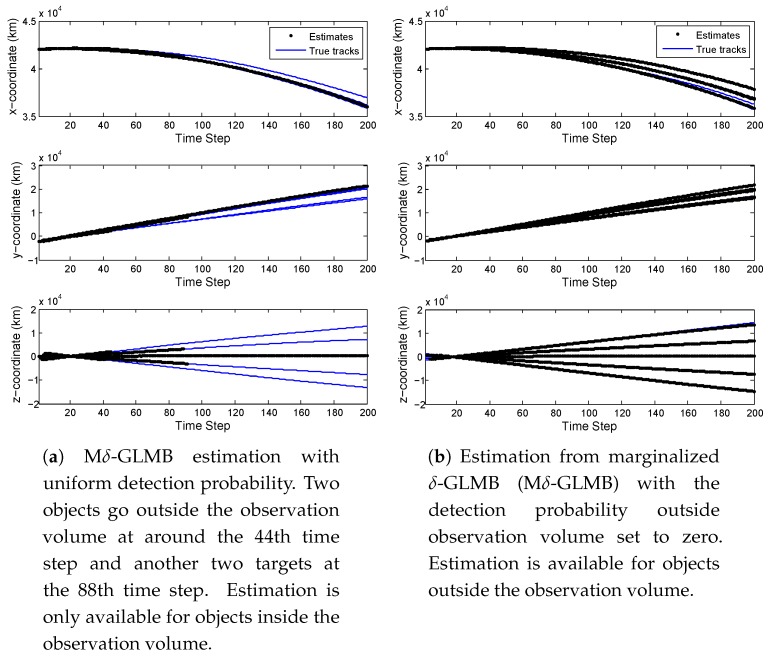
Estimation with different detection probability settings.

The estimation from the Mδ-GLMB filter with uniform detection probability [13,18,48,54] is shown in Figure 4a. The filtering with uniform detection probability cannot provide estimation for targets when they are outside of the observation volumes. The estimation from Mδ-GLMB with detection probability outside of the observation volume set to zero is shown in Figure 4b.

Since this section is intended to show the estimation of target positions, estimation results from one simulation are shown here, instead of the mean values from all simulations. Note that all the simulations had similar results. It can be seen from Figure 4b that even when the measurements for objects outside the observation volume were not available, estimation could still be provided. In this case, the estimation for objects outside was essentially the prediction. The figure also demonstrates that the estimation became worse for objects outside the observation volume since there were no measurements to update.

### 7.2. Consensus among Nodes with Different Observation Volumes

This subsection demonstrates the effectiveness of information fusion with a consensus algorithm for nodes with different observation volumes. It is complicated to fuse information from nodes with no estimation for objects outside the observation volumes. The fusion algorithm must consider the observation volume overlap, which makes the fusion more difficult when more nodes are involved. With the estimation available for objects outside the observation volume, the fusion algorithm can be performed in the same way as for nodes with the same observation volume.

Five nodes in the same position as node1 with complementary observation volumes are used in this subsection. The observation volumes for nodes were, respectively,

$$\begin{array}{ccc} V1 & = & \left[30,000\;\mathrm{km}\;\;\;40,000\;\mathrm{km}\right]\;\times \left[-15^{\circ }\;\;0^{\circ }\right]\;\times \left[-5^{\circ }\;\;5^{\circ }\right],\\ V2 & = & \left[30,000\;\mathrm{km}\;\;\;40,000\;\mathrm{km}\right]\;\times \left[-15^{\circ }\;\;0^{\circ }\right]\;\times \left[5^{\circ }\;\;15^{\circ }\right],\\ V3 & = & \left[30,000\;\mathrm{km}\;\;\;40,000\;\mathrm{km}\right]\;\times \left[-15^{\circ }\;\;0^{\circ }\right]\;\times \left[15^{\circ }\;\;25^{\circ }\right],\\ V4 & = & \left[30,000\;\mathrm{km}\;\;\;40,000\;\mathrm{km}\right]\;\times \left[-15^{\circ }\;\;0^{\circ }\right]\;\times \left[-15^{\circ }\;\;-5^{\circ }\right],\\ V5 & = & \left[30,000\;\mathrm{km}\;\;\;40,000\;\mathrm{km}\right]\;\times \left[-15^{\circ }\;\;0^{\circ }\right]\;\times \left[-25^{\circ }\;\;-15^{\circ }\right]. \end{array}$$

**Figure 5:**
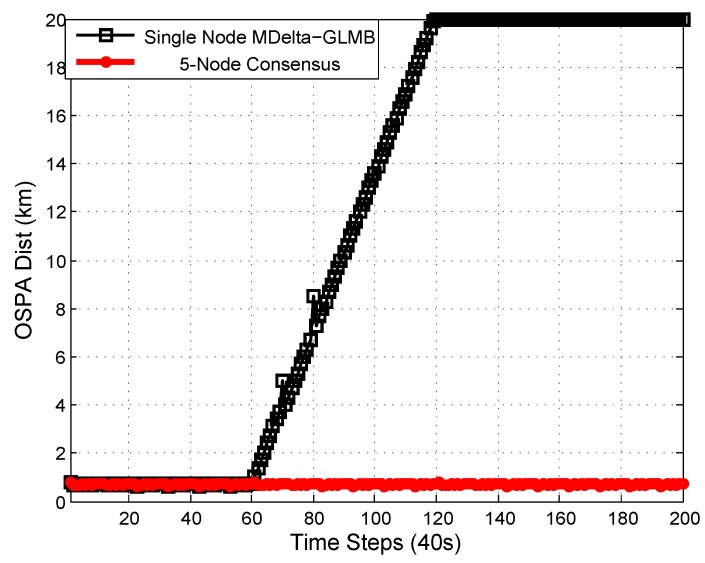
Estimation from single-node and 5-node consensus. Objects are starting to go outside the observation volume from the 60th time step and the performance for a single node becomes worse. Five-node consensus with complementary observation volumes provides accurate estimation for objects as long as they are in at least one node’s observation volume.

**Figure 6:**
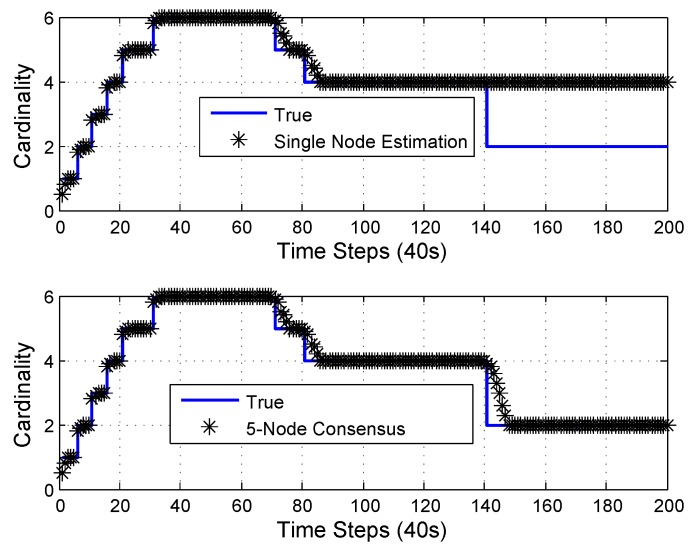
Cardinality performance from single-node and 5-node consensus. Two targets die at the 140th time step. A single node cannot provide cardinality estimation for objects outside the observation volume, while multiple nodes can be used to counteract the lack of observation volume of a single node.

This ensures that all objects were inside the combined observation volume of all sensors. The OSPA performance of a single-node and 5-node consensus is shown in Figure 5. The cardinality performance of a single-node and 5-node consensus is shown in Figure 6.

It can be seen that the single-node Mδ-GLMB filter [48] could provide good estimation for objects inside the observation volume. Since there was no update for objects outside the observation volume and only prediction was available, the estimation became worse with time. Two objects disappeared at the 140th time step, at which time they were outside the observation volume. A single node could not provide accurate cardinality estimation for objects outside the observation volume. Since all objects were in the combined observation volume of five nodes all the time, information fusion from five nodes could provide better estimation than a single node for objects outside the observation volume.

Since there was no measurement outside the observation volume, the best we could do is use the prediction model as the final estimation. The estimation performance was subject to the dynamic model which was characterized by the transition density noise. As time progressed, the estimation error became larger and larger without bound until new measurements were captured. The estimation confidence can be illustrated by the covariance matrix.

### 7.3. Consensus for Nodes with Similar Observation Volumes

**Figure 7:**
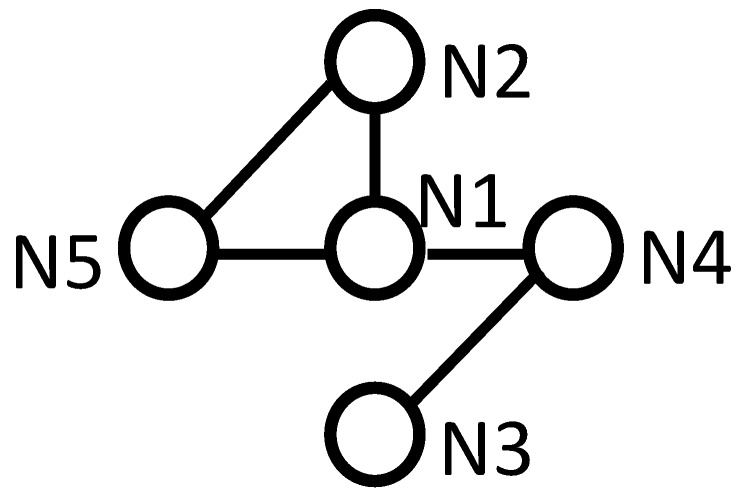
Network topology. Note that this topology is used as an example to show the efficacy of the consensus algorithm. The topology can be time-varying and unknown.

This subsection is to show the performance of the consensus algorithm for nodes with similar observation volumes. The angle range of all nodes was $50^{\circ }$, which was large enough for all objects to stay in the observation volume of all nodes all the time. The connection among the nodes is shown in Figure 7. A network with only five nodes was used in this paper to show the efficacy of the proposed method in distributed space debris tracking. A larger-scale network would involve more computation and processing time.

We only show the estimation from node3.

**Figure 8:**
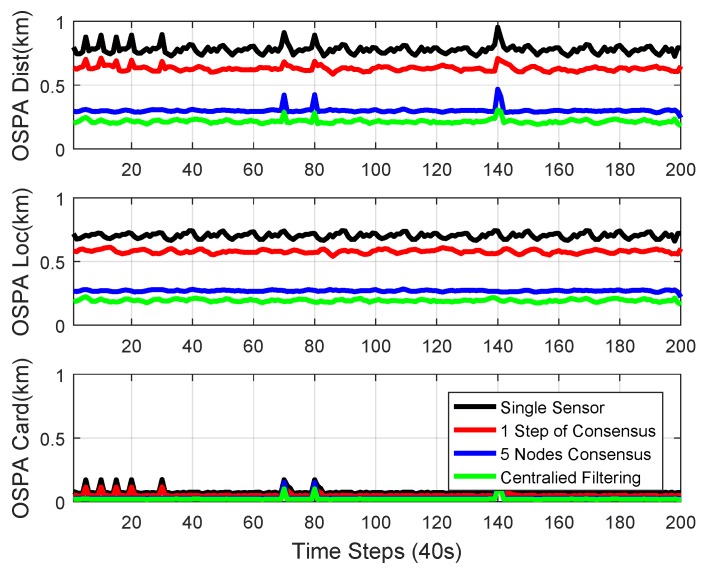
Optimal sub-pattern assignment (OSPA) performance from single sensor, 1-step consensus, 5-node consensus, and centralized Mδ-GLMB filtering.

The numerical experimental results from a single sensor [48], 1-step consensus, 5-node consensus, and centralized Mδ-GLMB filtering [48] are shown in Figure 8. Five-node consensus was the fusion procedure for all the information from five nodes. It is shown that the performance of 1-step consensus was better than single-node estimation. Since 5-node consensus uses information from all the nodes, it had better performance than 1-step consensus. Centralized Mδ-GLMB filtering makes use of raw measurements from all sensors, and had the best performance.

**Figure 9:**
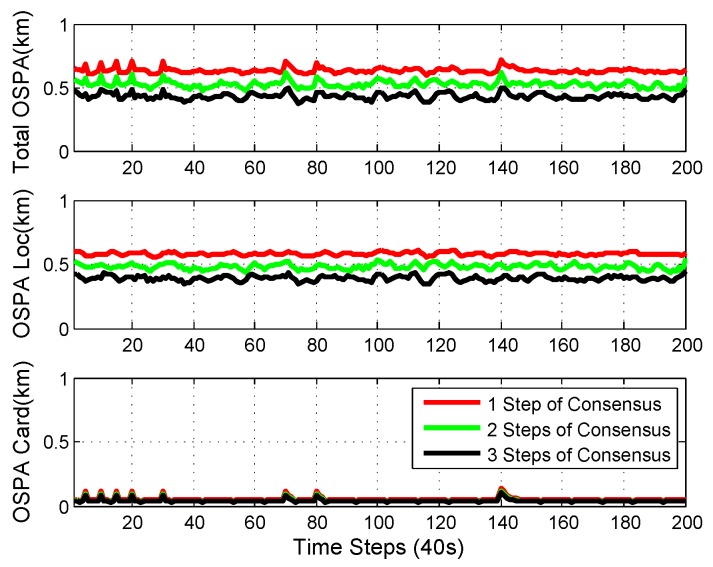
OSPA performance with different consensus steps. More consensus steps yielded better performance.

OSPA performance with different consensus steps is shown in Figure 9. It is shown in the figure that the OSPA performance was better with more consensus steps. With the designed network topology, the 3-step consensus had similar performance to the 5-node consensus. This is because it took 3 steps at most to transfer information from all of the other nodes to node3. After 3 steps of consensus, every node in the network had similar information, which is the average of the information from all nodes. There was no central node and the processing for each node was only carried among its neighbors, which makes the fusion scalable with respect to the size of the network.

**Figure 10:**
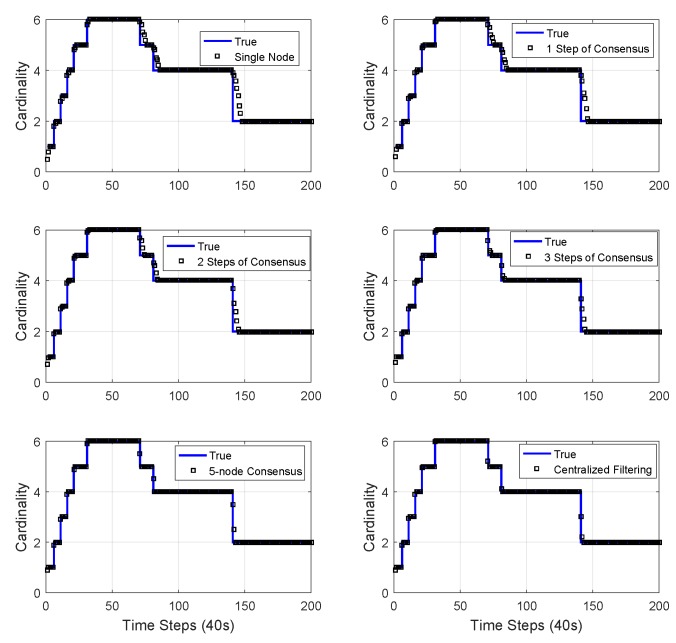
The mean of the estimated cardinality of single-node Mδ-GLMB, 1-step consensus, 2-step consensus, 3-step consensus, 5-node consensus, and centralized Mδ-GLMB filtering versus time.

**Figure 11:**
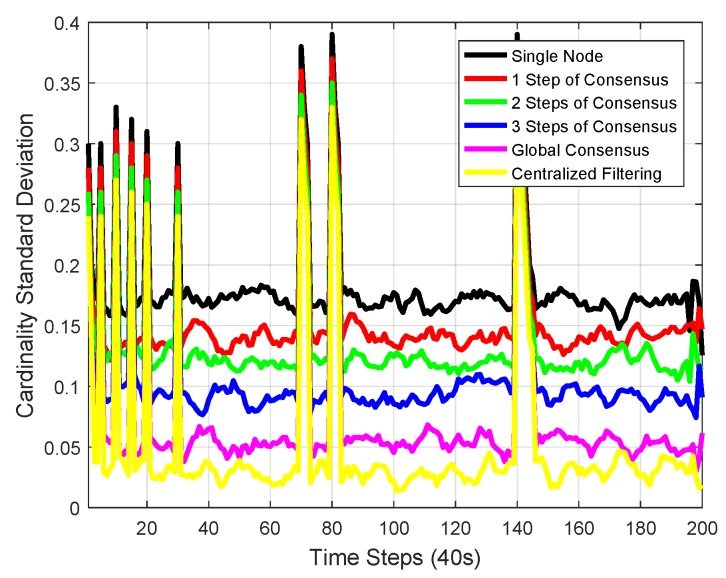
The standard deviation of the estimated cardinality of single-node Mδ-GLMB, 1-step consensus, 2-step consensus, 3-step consensus, 5-node consensus, and centralized Mδ-GLMB filtering versus time.

The mean and standard deviation of the estimated cardinality of single-node Mδ-GLMB, 1-step consensus, 2-step consensus, 3-step consensus, 5-node consensus, and centralized Mδ-GLMB filtering versus time are shown in Figure 10 and Figure 11, respectively. All filters estimated the cardinality accurately. The centralized Mδ-GLMB filtering had the best cardinality estimation performance. Consensus with more steps showed better cardinality estimation.

We can see that there are peaks in the plots in Figure 8, Figure 9 and Figure 11. The peaks happen at the time when there was a change in the number of objects. The change in the number of objects also contributed to the peaks in the cardinality standard deviation estimation. Cardinality estimation had a larger error than when the number of objects was not changing. The peaks in the total OSPA distance are because of the peaks in the cardinality OSPA distance.

**Figure 12:**
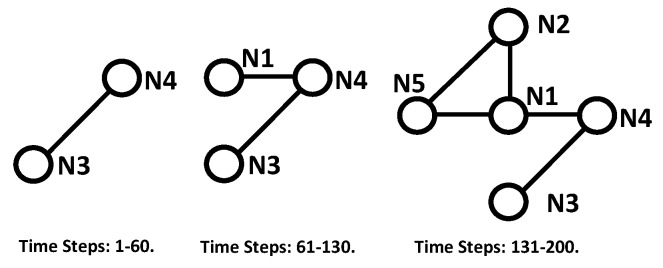
The time-varying topology for different time steps.

**Figure 13:**
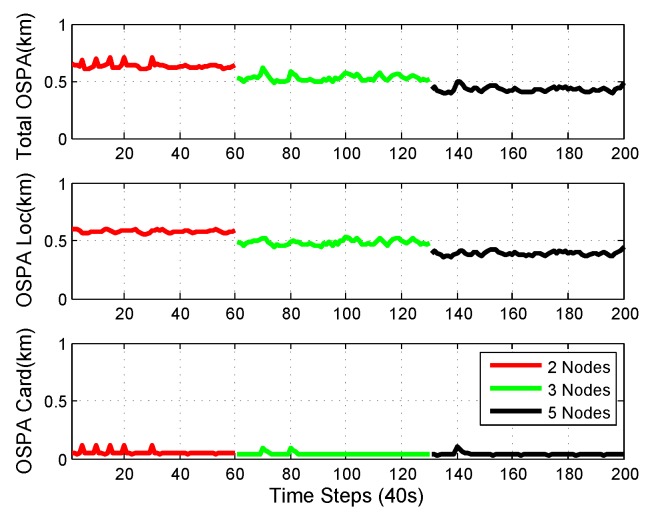
OSPA performance for time-varying topology.

The network can also be time-varying because the processing is performed only among neighbors. The time-varying topology is shown in Figure 12, and the performance of the consensus algorithm is shown in Figure 13. Node3 and node4 were in the network from the start to the 60th time step. Node3, node4, and node1 were in the network from the 61st time step to the 130th time step. All five nodes were in the network the rest of the time. Carrying out consensus only among a node’s neighbors makes it possible for the method to handle a network topology that is time-varying.

## 8. Concluding Remarks

This paper presented a consensus algorithm for space debris tracking with labeled RFS. The key innovation lies in the fusion among sensors with different observation volumes, and most importantly, the processing load for each node is scalable with respect to the size of the network. The system is robust because of the avoidance of data incest. A salient feature is that the network topology can be time-varying. The scalability makes the approach appealing for future space object tracking systems as more nodes are connected to the network. Experiments confirm that the proposed algorithm has the potential to be used in distributed space debris tracking systems.

In recent years, there is more need for finite-time average consensus algorithms. Future work will focus on distributed space debris tracking systems with finite-time average consensus methods. Strengths and shortcomings of different consensus algorithms will be presented for different tracking scenarios in future work.

## Figures and Tables

**Table 1:** State propagation of space debris.

•	input: {$\omega_{i},m_{i},P_{i}{\}}_{i=1}^{N}$
•	output: {$\omega_{i}^{+},m_{i}^{+},P_{i}^{+}{\}}_{i=1}^{N}$
for	i=1:N
	(1) 2n+1 weighted sigma points $S^{\left(j\right)}=\left\{\left(W^{\left(j\right)},\chi^{\left(j\right)}\right)\right\}$ are chosen
	to represent the Gaussian.
	(2) every sigma point is propagated with the help of Turboprop.
	(3) the predicted Gaussian is reconstructed with the predicted sigma points.
end	

## References

[1] Bobrinsky N., Del Monte L. (2010). The space situational awareness program of the European Space Agency. Cosm. Res..

[2] Talent D., Vilas F. A space-based concept for a collision warning sensor. Proceedings of the Space Programs and Technologies Conference.

[3] Jones B.A., Doostan A. (2013). Satellite collision probability estimation using polynomial chaos expansions. Adv. Space Res..

[4] Jones B.A., Vo B.N. A labeled multi-Bernoulli filter for space object tracking. Proceedings of the 2014 AAS/AIAA Spaceflight Mechanics Meeting.

[5] Jones B.A., Doostan A., Born G.H. (2013). Nonlinear propagation of orbit uncertainty using non-intrusive polynomial chaos. J. Guid. Control Dyn..

[6] National Research Council (2012). Continuing Kepler’s Quest: Assessing Air Force Space Command’s Astrodynamics Standards.

[7] Brooks R.R., Ramanathan P., Sayeed A.M. (2003). Distributed target classification and tracking in sensor networks. Proc. IEEE.

[8] Zou Y., Chakrabarty K. (2007). Distributed mobility management for target tracking in mobile sensor networks. IEEE Trans. Mob. Comput..

[9] Olfati-Saber R., Murray R.M. (2004). Consensus problems in networks of agents with switching topology and time-delays. IEEE Trans. Autom. Control.

[10] Zhu S., Chen C., Li W., Yang B., Guan X. (2013). Distributed optimal consensus filter for target tracking in heterogeneous sensor networks. IEEE Trans. Cybern..

[11] Olfati-Saber R., Fax J.A., Murray R.M. (2007). Consensus and cooperation in networked multi-agent systems. Proc. IEEE.

[12] Kamal A.T., Farrell J.A., Roy-Chowdhury A.K. Information consensus for distributed multi-target tracking. Proceedings of the IEEE Conference on Computer Vision and Pattern Recognition.

[13] Battistelli G., Chisci L., Fantacci C., Farina A., Graziano A. (2013). Consensus CPHD filter for distributed multitarget tracking. IEEE J. Sel. Top. Signal Process..

[14] Olfati-Saber R. Kalman-consensus filter: Optimality, stability, and performance. Proceedings of the 48th IEEE Conference on Decision and Control (CDC) Held Jointly with 2009 28th Chinese Control Conference.

[15] Saber R.O., Murray R.M. Consensus protocols for networks of dynamic agents. Proceedings of the 2003 American Control Conference.

[16] Olfati-Saber R. Distributed Kalman filtering for sensor networks. Proceedings of the 2007 46th IEEE Conference on Decision and Control.

[17] Olfati-Saber R., Shamma J.S. Consensus filters for sensor networks and distributed sensor fusion. Proceedings of the 44th IEEE Conference on Decision and Control.

[18] Fantacci C., Vo B.-N., Vo B.-T., Battistelli G., Chisci L. (2018). Robust fusion for multisensor multiobject tracking. IEEE Signal Process. Lett..

[19] Battistelli G., Chisci L. (2014). Kullback–Leibler average, consensus on probability densities, and distributed state estimation with guaranteed stability. Automatica.

[20] Mahler R.P. (2007). Statistical Multisource-Multitarget Information Fusion.

[21] Mahler R.P. (2014). Advances in Statistical Multisource-Multitarget Information Fusion.

[22] Mahler R.P. (2003). Multitarget Bayes Filtering via First-Order Multitarget Moments. IEEE Trans. Aerosp. Electron. Syst..

[23] Vo B.N., Ma W.K. (2006). The Gaussian mixture probability hypothesis density filter. Trans. Signal Process..

[24] Mahler R. (2007). PHD filters of higher order in target number. IEEE Trans. Aerosp. Electron. Syst..

[25] Vo B.T., Vo B.N., Cantoni A. (2007). Analytic implementations of the cardinalized probability hypothesis density filter. IEEE Trans. Signal Process..

[26] Vo B.T., Vo B.N., Cantoni A. (2009). The cardinality balanced multi-target multi-Bernoulli filter and its implementations. IEEE Trans. Signal Process..

[27] Vo B.N., Vo B.T., Pham N.T., Suter D. (2010). Joint detection and estimation of multiple objects from image observations. IEEE Trans. Signal Process..

[28] Vo B.T., Vo B.N. (2013). Labeled random finite sets and multi-object conjugate priors. IEEE Trans. Signal Process..

[29] Vo B.N., Vo B.T., Phung D. (2014). Labeled random finite sets and the Bayes multi-target tracking filter. IEEE Trans. Signal Process..

[30] Vo B.N., Vo B.T., Hoang H. (2016). An Efficient Implementation of the Generalized Labeled Multi-Bernoulli Filter. IEEE Trans. Signal Process..

[31] Papi F., Vo B.N., Vo B.T., Fantacci C., Beard M. (2015). Generalized Labeled Multi-Bernoulli Approximation of Multi-Object Densities. IEEE Trans. Signal Process..

[32] Reuter S., Vo B.T., Vo B.N., Dietmayer K. (2014). The labeled multi-Bernoulli filter. IEEE Trans. Signal Process..

[33] Faber W., Chakravorty S., Hussein I.I. A randomized sampling based approach to multi-object tracking. Proceedings of the 2015 18th International Conference on Information Fusion (Fusion).

[34] Hussein I., DeMars K., Früh C., Jah M., Erwin R. An AEGIS-FISST algorithm for multiple object tracking in space situational awareness. Proceedings of the AIAA/AAS Astrodynamics Specialist Conference.

[35] DeMars K.J., Hussein I.I., Frueh C., Jah M.K., Scott Erwin R. (2015). Multiple-Object Space Surveillance Tracking Using Finite-Set Statistics. J. Guid. Control Dyn..

[36] Faber W., Chakravorty S., Hussein I.I. (2016). Multi-Object Tracking with Multiple Birth, Death, and Spawn Scenarios Using A Randomized Hypothesis Generation Technique (R-FISST). arXiv.

[37] Hussein I., Frueh C., Erwin R., Jah M. An AEGIS-FISST algorithm for joint detection, classification, and tracking. Proceedings of the 2013 AAS/AIAA Astrodynamics Specialist Conference.

[38] Hussein I.I., DeMars K.J., Früh C., Erwin R.S., Jah M.K. An AEGIS-FISST integrated detection and tracking approach to Space Situational Awareness. Proceedings of the 2012 15th International Conference on Information Fusion.

[39] DeMars K.J., Hussein I.I., Jah M.K., Erwin R.S. The Cauchy-Schwarz divergence for assessing situational information gain. Proceedings of the 2012 15th International Conference on Information Fusion, Information Fusion (FUSION).

[40] Wei B., Nener B. Consensus labeled multi-Bernoulli filtering for distributed space debris tracking. Proceedings of the 2017 International Conference on Control, Automation and Information Sciences (ICCAIS).

[41] Jones B.A., Gehly S., Axelrad P. Measurement-based birth model for a space object cardinalized probability hypothesis density filter. Proceedings of the AIAA/AAS Astrodynamics Specialist Conference, AIAA SPACE Forum, (AIAA 2014-4311).

[42] Wei B., Nener B., Liu W., Ma L. (2017). Global Tracking of Space Debris via CPHD and Consensus. Adv. Space Res..

[43] Hill K., Jones B. (2009). TurboProp Version 4.0..

[44] Julier S.J., Uhlmann J.K. New extension of the Kalman filter to nonlinear systems. Proceedings of the Signal Processing, Sensor Fusion, and Target Recognition VI.

[45] Schwarz G. (1978). Estimating the Dimension of a Model. Ann. Stat..

[46] Calafiore G.C., Abrate F. (2009). Distributed linear estimation over sensor networks. Int. J. Control.

[47] Mahalanobis P.C. (1925). Analysis of Race-Mixture in Bengal.

[48] Fantacci C., Vo B.T., Papi F., Vo B.N. (2015). The Marginalized delta-GLMB Filter. arXiv.

[49] Chopra N., Spong M.W. (2008). Output synchronization of nonlinear systems with relative degree one. Recent Advances in Learning and Control.

[50] Li Z., Duan Z., Chen G., Huang L. (2010). Consensus of multiagent systems and synchronization of complex networks: A unified viewpoint. IEEE Trans. Circuits Syst. I Regul. Pap..

[51] Xiao F., Wang L. (2006). State consensus for multi-agent systems with switching topologies and time-varying delays. Int. J. Control.

[52] Tsitsiklis J.N. (1984). Problems in Decentralized Decision Making and Computation.

[53] Jones B.A., Bryant D.S., Vo B.T., Vo B.N. Challenges of multi-target tracking for space situational awareness. Proceedings of the 2015 18th International Conference on Information Fusion (Fusion).

[54] Fantacci C. (2015). Distributed multi-object tracking over sensor networks: A random finite set approach. arXiv.

[55] DeMars K.J., McCabe J.S., Darling J.E. Collaborative multi-sensor tracking and data fusion. Proceedings of the 5th AAS/AIAA Space Flight Mechanics Meeting.

[56] Wang B., Yi W., Li S., Morelande M.R. Distributed multi-target tracking via generalized multi-Bernoulli random finite sets. Proceedings of the 2015 18th International Conference on Information Fusion (Fusion).

[57] Oliva G., Setola R., Hadjicostis C.N. (2016). Distributed Finite-Time Average-Consensus with Limited Computational and Storage Capability. IEEE Trans. Control Netw. Syst..

[58] Hendrickx J.M., Shi G., Johansson K.H. (2015). Finite-Time Consensus Using Stochastic Matrices with Positive Diagonals. IEEE Trans. Autom. Control.

[59] Faramondi L., Setola R., Oliva G. (2018). Performance and robustness of discrete and finite time average consensus algorithms. Int. J. Syst. Sci..

[60] Sundaram S., Hadjicostis C.N. Finite-Time Distributed Consensus in Graphs with Time-Invariant Topologies. Proceedings of the 2007 American Control Conference.

[61] Hendrickx J.M., Jungers R., Olshevsky A., Vankeerberghen G. (2014). Graph diameter, eigenvalues, and minimum-time consensus. Automatica.

[62] Katragadda S., Sanmiguel J.C., Cavallaro A. Consensus protocols for distributed tracking in wireless camera networks. Proceedings of the 17th International Conference on Information Fusion (FUSION).

[63] Schuhmacher D., Vo B.T., Vo B.N. (2008). A consistent metric for performance evaluation of multi-object filters. IEEE Trans. Signal Process..

